# Downregulation of glutamic acid decarboxylase in Drosophila TDP-43-null brains provokes paralysis by affecting the organization of the neuromuscular synapses

**DOI:** 10.1038/s41598-018-19802-3

**Published:** 2018-01-29

**Authors:** Giulia Romano, Nikola Holodkov, Raffaella Klima, Federica Grilli, Corrado Guarnaccia, Monica Nizzardo, Federica Rizzo, Rodolfo Garcia, Fabian Feiguin

**Affiliations:** 1 International Centre for Genetic Engineering and Biotechnology, Padriciano 99, 34149 Trieste, Italy; 2 Department of Pathophysiology and Transplantation (DePT), Dino Ferrari Centre, University of Milan, Neuroscience Section, IRCCS Foundation Ca’ Granda Ospedale Maggiore Policlinico, Via Francesco Sforza 35, 20122 Milan, Italy

## Abstract

Amyotrophic lateral sclerosis is a progressive neurodegenerative disease that affects the motor system, comprised of motoneurons and associated glia. Accordingly, neuronal or glial defects in TDP-43 function provoke paralysis due to the degeneration of the neuromuscular synapses in Drosophila. To identify the responsible molecules and mechanisms, we performed a genome wide proteomic analysis to determine differences in protein expression between wild-type and TDP-43-minus fly heads. The data established that mutant insects presented reduced levels of the enzyme glutamic acid decarboxylase (Gad1) and increased concentrations of extracellular glutamate. Genetic rescue of Gad1 activity in neurons or glia was sufficient to recuperate flies locomotion, synaptic organization and glutamate levels. Analogous recovery was obtained by treating TDP-43-null flies with glutamate receptor antagonists demonstrating that Gad1 promotes synapses formation and prevents excitotoxicity. Similar suppression of TDP-43 provoked the downregulation of GAD67, the Gad1 homolog protein in human neuroblastoma cell lines and analogous modifications were observed in iPSC-derived motoneurons from patients carrying mutations in TDP-43, uncovering conserved pathological mechanisms behind the disease.

## Introduction

Amyotrophic lateral sclerosis (ALS) affects motoneuron performance leading to muscles denervation, wasting and paralysis. Although the pathological origin of the disease is not well known, defects in the solubility and intracellular distribution of the ribonuclear protein (RNP) TDP-43 strongly correlate with the neurological symptoms and histological modifications observed in the great majority of affected patients^1–3^. Several experimental manipulations have been performed in different animal models, as mouse^4–7^, *C.elegans*^8,9^, *D.rerio*^10–12^ as well as in *D.melanogaster*^13–16^, which have clearly demonstrated that the suppression of TDP-43 function is sufficient to reproduce the main characteristics of the disease: locomotive defects, neurodegeneration and reduced life span. This has raised the issue of TDP-43 playing a central role in the pathophysiological mechanisms of ALS. In this direction, we have recently described the initial events that lead to muscle denervation and motoneuron degeneration upon the acute suppression of endogenous TDP-43 protein (TBPH) in Drosophila *melanogaster* neurons or glial cells^17,18^. Therefore, we found that the neuronal or glial function of TBPH was similarly required to prevent locomotive alterations and preserve the postsynaptic organization of the glutamate receptors (GluRIIA) present at the neuromuscular junctions (NMJs) of flies. These results support the idea that ALS could present a non-neuronal origin and, implies that alterations in TBPH function inside neurons or glial cells may contribute to the disease by affecting the regulations of analogous metabolic pathways^13,19,20^. In order to test this hypothesis and identify those molecules, we decided to perform a genome wide high throughput proteomic analysis by combining high-resolution two-dimensional (2D) gel electrophoresis with MALDI-TOF mass spectrometry. We reasoned that this approach would succeed in identifying mRNA target molecules regulated by TBPH at both translational or post-transcriptional level, as recently suggested for the conserved microtubule binding protein *futsch*/MAP1B^21^. The present study reports that the enzyme glutamic acid decarboxylase 1 (Gad1) appears downregulated in TBPH mutant brains and describes that Gad1 function is required in neurons or non-autonomously in glial tissues. It promotes motoneuron synaptogenesis and muscle innervation through the correct organization of the glutamate receptors at the postsynaptic membranes, more specifically by limiting an excessive accumulation of the glutamate neurotransmitter in the extracellular space. We also show that the TBPH-Gad1 regulatory relationship is conserved in human neuronal cell lines and is present in pluripotent stem cells derived from ALS patients carrying mutations in TDP-43. The data obtained provide novel bases to understand the pathological process behind the disease and open the way to novel therapeutic interventions.

## Results

### Proteomic analysis identifies reduced levels of Gad1 in TBPH null brains

**Figure 1:**
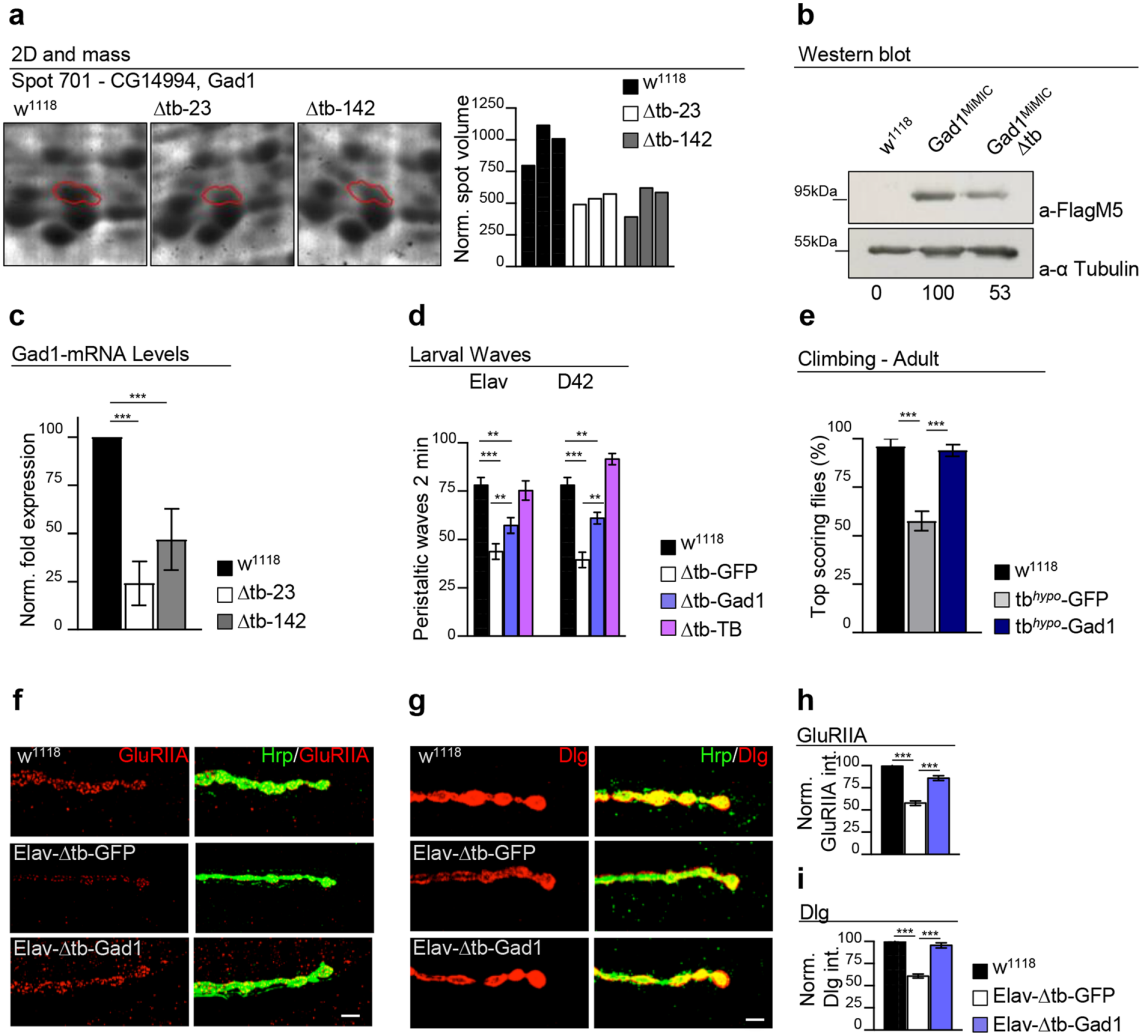
Gad1 is downregulated in TBPH-mutant flies and required for synaptogenesis. (**a**) Two-dimensional electrophoresis analysis of adult heads in Drosophila revealed that the spot 701, corresponding to the Gad1-CG14994 protein was reduced in Δtb-23 and Δtb-142 (tbph^Δ23/Δ23^ and tbph^Δ142/Δ142^) null alleles compared to w^1118^controls. The bar graph indicates quantified spot intensities (volumes), after gel normalization. *n* = 3. (**b**) Western blot analysis of w^1118^, Gad1^MiMIC^ (w; +/+;Mi{MIC}Gad1^MI09277^/+) and Gad1^MiMIC^ − Δtb (w;tbph^Δ23^/tbph^Δ23^;Mi{MIC}Gad1^MI09277^/+). Third instar larval brains were probed with anti-FlagM5 and alpha-tubulin antibodies. The same membrane was probe with the two antibodies and the bands of interest were cropped. Quantification of normalized amounts was reported below each lane. *n* = 3. (**c**) Real time PCR of *Gad1* transcript levels normalized on *Rpl11* (housekeeping) in third instar larval brains of w^1118^, Δtb-23 (tbph^Δ23/Δ23^) and Δtb-142 (tbph^Δ142/Δ142^). *n* = 3. (**d**) Number of peristaltic waves between w^1118^, Δtb-GFP (*left:* elav-GAL4,tbph^Δ23^/tbph^Δ23^;UAS-GFP/ +; *right:* tbph^Δ23^/tbph^Δ23^;D42-GAL4/UAS-GFP), Δtb-Gad1 (*left side:* elav-GAL4,tbph^Δ23^/tbph^Δ23^;UAS-Gad1/+; *right side:* tbph^Δ23^/tbph^Δ23^;D42-GAL4/UAS-Gad1) and Δtb-TB (*left side:* elav-GAL4,tbph^Δ23^/tbph^Δ23^,UAS-TBPH; *right side:* tbph^Δ23^/tbph^Δ23^,UAS-TBPH;D42-GAL4/+). *n* = 25. (**e**) Climbing assay on 4 days old adult flies compare w^1118^, tb^hypo^-GFP (elav-GAL4,tbph^Δ23^/UAS-GFP;UAS-*Dcr-2*,TBPH-RNAi/+) and tb^hypo^-Gad1 (elav-GAL4,tbph^Δ23^/ + ;UAS-*Dcr-2*,TBPH-RNAi/UAS-Gad1). *n* = 100. (**f**) Confocal images of third instar NMJ terminals, muscle 6/7 second segment stained with anti-HRP (in green) and anti-GluRIIA (in red) in w^1118^, Elav-Δtb-GFP and Elav-Δtb-Gad1. (**g**) Confocal images of third instar NMJ terminals in muscle 6/7 second segment stained with anti-HRP (in green) and anti-Dlg (in red) in w^1118^, Elav-Δtb-GFP and Elav-Δtb-Gad1. (**h-i**) Quantification of GluRIIA intensity and Dlg intensity, *n* > 200 boutons. **p < 0.01, ***p < 0.001 calculated by one-way ANOVA. Error bars SEM. Scale bar 5 µm.

Since mRNA levels are not necessarily related to actual protein expression, we decided to use two-dimensional gel electrophoresis to establish protein levels in wild-type vs TBPH minus flies. This approach could result in the identification of new molecules potentially responsible for the neurological phenotypes described in TBPH minus flies. Protein 2D maps of 1 day-old wild-type and TBPH null adult Drosophila heads were analyzed after normalization for statistical differences in spot intensities. The number of spots modified by 1.5 > fold was 35/1383 (Supplementary Fig. S1a–d). Among them we focused on those found modified in the same direction for both mutant alleles (tbph^Δ23^ and tbph^Δ142^) compared with the wild type flies. We also excluded spots in gel areas of heavy streaking and/or abnormally strong background making them unreliable, after which a total of 5 down-regulated and 2 up-regulated spots were subjected to mass spectrometry identification (Supplementary Fig. S1d and Table 1). We found that one of the most significantly down-regulated spots in mutant heads corresponded to the protein glutamic acid decarboxylase Gad1 (CG14994, FBgn0004516) (Fig. 1a). This enzyme is very well conserved in mammals and its catalytic function is required in Drosophila embryos to regulate extracellular levels of the neurotransmitter glutamate and define the postsynaptic organization of the glutamatergic synapses present at the neuromuscular junctions (NMJ)^22^. Since antibodies anti-Gad1 are not available, the proteomic results were validated with an alternative immunodetection-based methodology. We utilized an allele of Gad1 endogenously tagged with EGFP after the insertion of the MiMIC cassette within its coding sequence^23–25^. Western blot analysis of Gad1^MiMIC^ brains (w;Mi{MIC}Gad1^MI09277^/+) using antibodies against flag showed the presence of a specific band of 95 kDa. This molecular mass is consistent with the sum of Gad1 (57.8 kDa) plus the EGFP-FlAsH-StrepII-TEV-3xFlag-tag insertion (35 kDa). The 95 kDa band was found to be clearly reduced in a TBPH minus background (tbph^Δ23^/tbph^Δ23^;Mi{MIC}Gad1^MI09277^/+) (Fig. 1b and Supplementary Fig. S6a). In support of the proteomic data, quantitative PCR assays revealed that the levels of Gad1 mRNA were also reduced in tbph^Δ23^ and tbph^∆142^ loss-of-function alleles (Fig. 1c). Therefore, the downregulation of Gad1 in TBPH null heads was expressed at both transcriptional and translational levels.

**Table 1:** The 7 modified 2D spots identified in TBPH mutants compared to control.

Spot	Gene (*FlyBase*)	Protein	Score	Experimental/Theoretical Molecular weight (kDa)	N° of peptides detected	Fold change mut vs. control
701	CG14994	Glutamic acid decarboxylase	125	57.72/57.8	18	↓1.83
187	CG17285	Fat body protein1, isoA	200	119.59/119.7	40	↓4.52
324	CG17285	Fat body protein1, isoA	79	119.59/119.7	30	↓3.04
470	CG17285	Fat body protein1, isoA	135	119.59/119.7	19	↓1.99
216	CG2979	Yolk protein2	134	49.63/49.7	29	↓3.21
344	CG4904	Proteasome α6 subunit	153	31.04/31.1	28	↑4.41
851	CG4190	Heat shock gene 67Bc	113	22.17/22.2	4	↑2.56

### Genetic rescue of Gad1 activity in presynaptic neurons or motoneurons recovered flies motility and the postsynaptic organization of neuromuscular junctions

**Figure 2:**
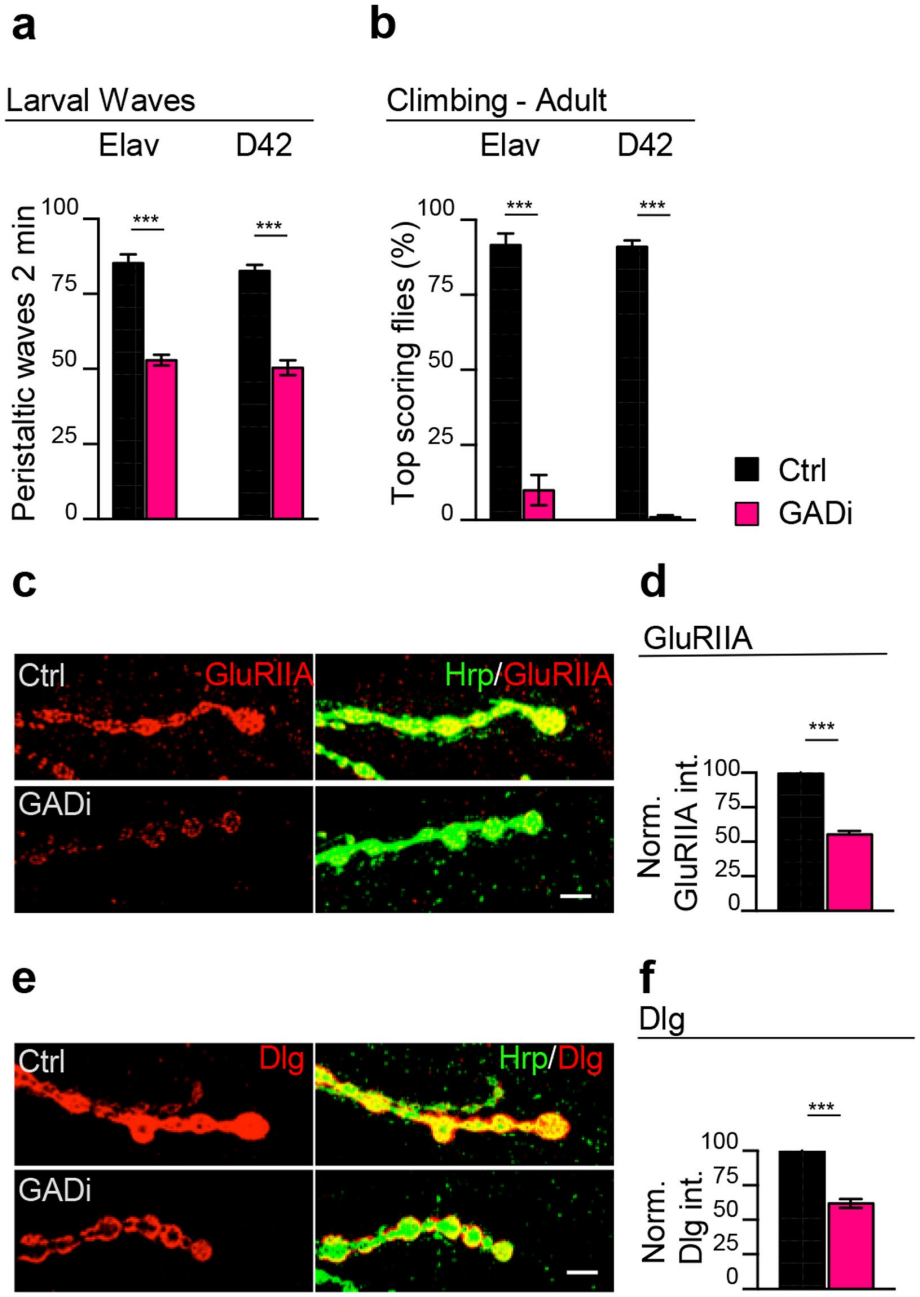
The reduction of Gad1 in neurons triggers postsynaptic assembly defects. (**a**) Number of peristaltic waves of Ctrl (*left side:* UAS-LacZ/elav-GAL4 and *right side:UAS-Dcr-2*/+;UAS-LacZ/+;D42-GAL4/+) and GADi (*left side:* elav-GAL4/+;GAD-RNAi/+ and *right side:* UAS-Dcr-2/+;;D42-GAL4/GAD-RNAi) larvae. *n* = 20. (**b**) Climbing assay on adult flies at day 4 of Ctrl and GADi on the left using elav-GAL4 and on the right using D42-GAL4. *n* = 100. (**c**) Confocal images of third instar NMJ terminals in muscle 6/7 second segment stained with anti-HRP (in green) and anti-GluRIIA (in red) in Ctrl and GADi (using elav-GAL4). (**d**) Quantification of GluRIIA intensity in Ctrl and GADi. *n* = 200 boutons. (**e**) Confocal images of third instar NMJ terminals in muscle 6/7 second segment stained with anti-HRP (in green) and anti-Dlg (in red) in Ctrl and GADi (using elav-GAL4). (**f**) Quantification of Dlg intensity in Ctrl and GADi. *n* = 200 boutons. ***p < 0.001, calculated by T-test. Error bars SEM. Scale bar 5 µm.

To test whether the downregulation of Gad1 played any role in TBPH loss-of-function phenotypes, we utilized the GAL4/UAS system to increase the expression of Gad1 in TBPH minus neurons using the specific driver elav-GAL4 (tbph^∆23/∆23^,elav-GAL4;UAS-Gad1). This strategy was sufficient to partially rescue the locomotive problems observed in TBPH minus third instar larvae (L3) and, more surprisingly, very efficiently recovered the TBPH minus motility defects after the expression of UAS-Gad1 in a more restricted group of cells like motoneurons using D42-GAL4 (tbph^∆23/∆23^;D42-GAL4/UAS-Gad1) (Fig. 1d). Despite the capacity of Gad1 to rescue larvae motility, we found that these larvae were not able to reach adult stages indicating that the overexpression of this transgene was not able to compensate the complete absence of TBPH. Therefore, to test whether the expression of Gad1 was also able to restore the normal locomotion of adult flies, similar rescue experiments were performed utilizing a hypomorphic allele of TBPH generated by the expression of an RNAi against this protein (tbph^∆23^,elav-GAL4/+;UAS-*Dcr-2*,TBPH-RNAi/UAS-Gad1). Thus, our experiments showed that the overexpression of Gad1 significantly enhances the climbing abilities of TBPH-hypomorphic flies compared to GFP-expressing controls (Fig. 1e and Supplementary Fig. S2h). At the molecular level, the neuronal expression of Gad1 in TBPH mutant larvae was sufficient to restore the wild-type organization of the glutamate receptors in postsynaptic clusters (GluRIIA) as well as the homogeneous localization of the disc-large protein (Dlg) around the presynaptic boutons (Fig. 1f–i). On the contrary, the neuronal expression of Gad1 was not able to promote motoneuron terminal growth or induce the formation of new presynaptic boutons (Supplementary Fig. S2a-g). Instead, the silencing of Gad1 in presynaptic neurons or solely in motoneurons (elav-GAL4/+;GAD-RNAi or UAS-Dcr-2/+;;D42-GAL4/GAD-RNAi) provoked strong locomotive defects and severe alterations in the intracellular organization of the postsynaptic proteins Dlg and GluRIIA (Fig. 2a–f and Supplementary Fig. S3a–f). This demonstrated that Gad1 plays a relevant role in the neurodegenerative process triggered by a TBPH dysfunction.

### Gad1 activity in the Drosophila glia is required for the postsynaptic clustering of glutamate receptors at the neuromuscular synapses

**Figure 3:**
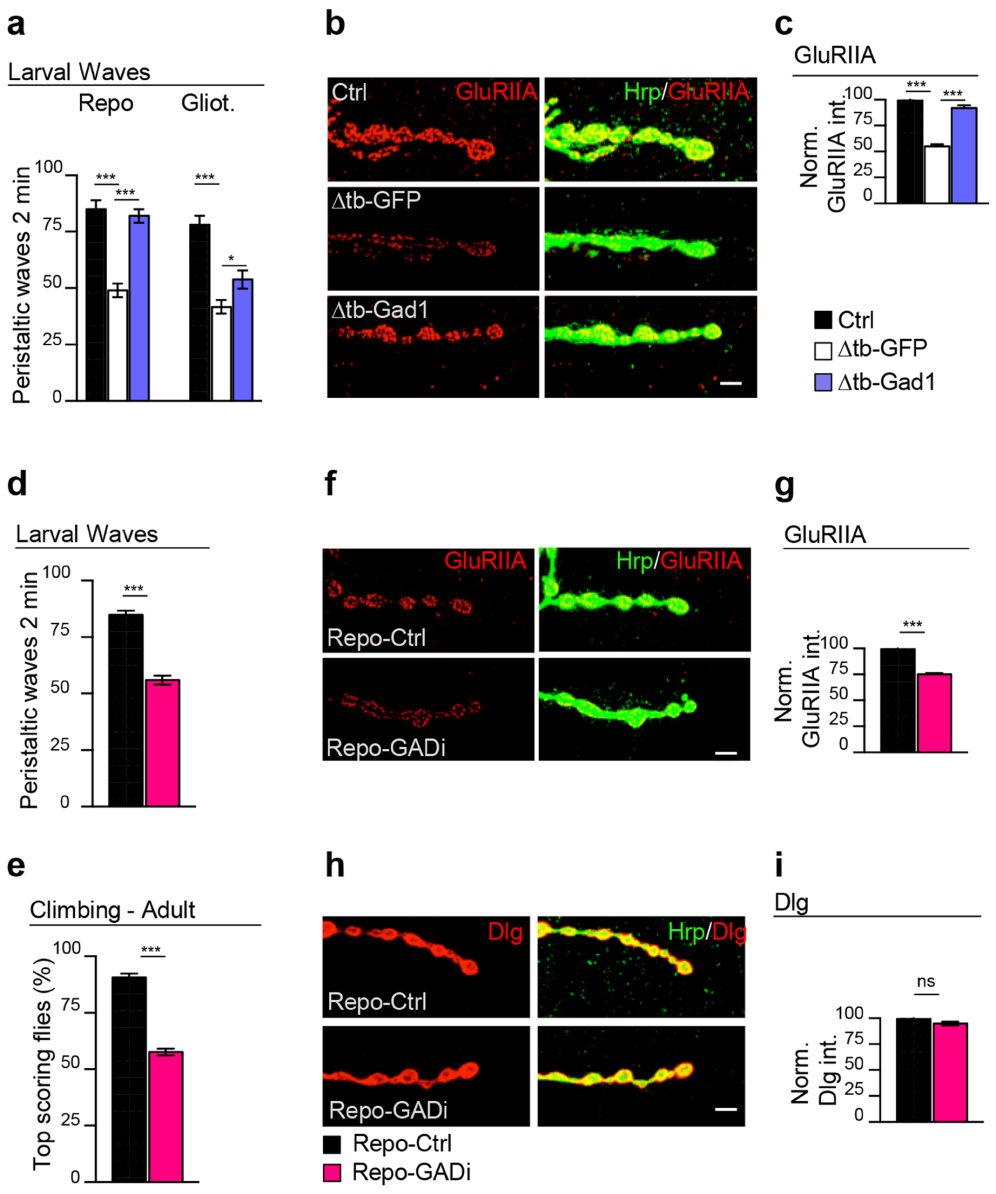
The glial activity of Gad1 is sufficient to ensure the postsynaptic clustering of the GluRIIA. (**a**) Number of peristaltic waves of Ctrl (*left side:* tbph^Δ23^/+; repo-GAL4,UAS-GFP/+; *right side:* tbph^Δ23^,gliotactin-GAL4/+; UAS-GFP/+), Δtb-GFP (*left side:* tbph^Δ23^/tbph^Δ23^;repo-GAL4/UAS-GFP; *right side:* tbph^Δ23^,gliotactin-GAL4/tbph^Δ23^;UAS-GFP/+) and Δtb-Gad1 (*left side:* tbph^Δ23^/tbph^Δ23^;repo-GAL4/UAS-Gad1; *left side:* tbph^Δ23^, gliotactin-GAL4/ tbph^Δ23^;UAS-GFP/+). *n* = 25. (**b**) Confocal images of third instar NMJ terminals in muscle 6/7 second segment stained with anti-HRP (in green) and anti-GluRIIA (in red) in Ctrl, Δtb-GFP and Δtb-Gad1 (using repo-GAL4). (**c**) Quantification of GluRIIA intensity. *n* > 200 boutons. (**d**) Number of peristaltic waves of Repo-Ctrl (UAS-*Dcr-2*/+;UAS-LacZ/ + ;repo-GAL4/+) and Repo-GADi (UAS-*Dcr-2*/+; +/+; repo-GAL4/GAD-RNAi) larvae. *n* = 20. (**e**) Climbing assay on adult flies at day 4 of Repo-Ctrl and Repo-GADi. *n* = 100. (**f**) Confocal images of third instar NMJ terminals in muscle 6/7 second segment stained with anti-HRP (in green) and anti-GluRIIA (in red) in Repo-Ctrl and Repo-GADi. (**g**) Quantification of GluRIIA intensity. *n* > 200 boutons. (**h**) Confocal images of third instar NMJ terminals in muscle 6/7 second segment stained with anti-HRP (in green) and anti-Dlg (in red) in Repo-Ctrl and Repo-GADi. (**i**) Quantification of Dlg intensity. *n* > 200 boutons. ns = not significant *p < 0.05, ***p < 0.001 calculated by one-way ANOVA in (a and c) and T-test in (d,e,g and i). Error bars SEM. Scale bar 5 µm.

Considering that the normal function of TBPH is also required in the Drosophila glia to prevent synaptic defects, we decided to analyze whether Gad1 activity was necessary in these tissues to prevent the neurological phenotypes described in TBPH minus flies^18^. Strikingly, we observed that the expression of Gad1 with the glial driver repo-GAL4 in TBPH minus flies (tbph^∆23/∆23^;repo-GAL4/UAS-Gad1) was able to completely restore the locomotive defects observed in L3 larvae (Fig. 3a). Interestingly, we found that the expression of Gad1 in the peripheral glia with gliotactin-GAL4^26,27^ significantly managed to recover the motility of the mutant flies indicating that the proper function of these specialized cells was sufficient to promote an amelioration of the peristaltic phenotypes. Anatomically, we found that the recovery of fly motility was accompanied by the re-formation of the glutamate receptor field at the postsynaptic neuromuscular synapses (Fig. 3b and c). In contrast, the expression of Gad1 was not able to regenerate the postsynaptic distribution of Dlg or induce the glial wrapping of neuromuscular axons (Supplementary Fig. S4a–d,f and i). At the presynaptic level, glial Gad1 expression was not able to promote the growth of motoneuron terminal axons or the recovery of Syntaxin (Supplementary Fig. S4e,g,h, j and k). In agreement with these results, we observed that the targeted suppression of Gad1 in the Drosophila glia using a specific RNAi under repo-GAL4 (UAS-Dcr-2/+;;repo-GAL4/GAD-RNAi) provoked serious locomotive problems and strongly disrupted the postsynaptic distribution of the glutamate receptors at the NMJ (Fig. 3d–g). The intracellular localization of Dlg instead, seemed not to be affected by the glial absence of Gad1 (Fig. 3h and i). Identically, the silencing of Gad1 did not affect motoneuron axon growth or presynaptic levels of the transmembrane proteins Syntaxin (Syx) or Bruchpilot (Brp) (Supplementary Fig. S4l–q) revealing an unexpected role of Gad1 in the glia, i.e. supporting synaptogenesis.

### TBPH minus flies present neurotoxic levels of extracellular glutamate

**Figure 4:**
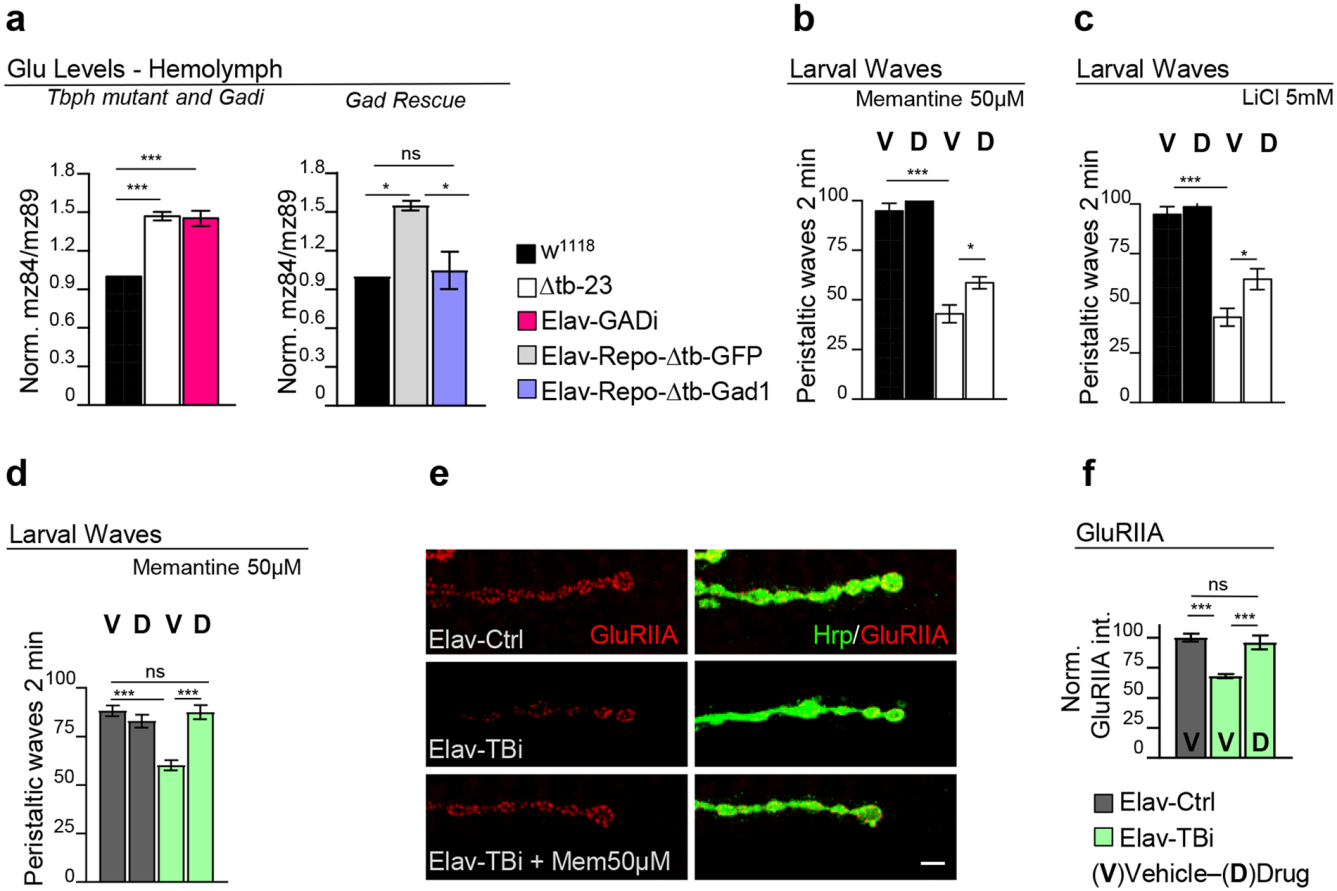
Gad1 downregulation provokes glutamate excitotoxicity in TBPH mutant flies. (**a**) Glutamate relative concentration in the hemolymph of third instar larvae: on the left graph in w^1118^, ∆tb-23 (tbph^Δ23/Δ23^) and Elav-GADi (elav-GAL4/+;GAD-RNAi/+) and on the right graph the glutamate levels in w^1118^, Elav-Repo-ΔtbGFP (elav-GAL4,tbph^Δ23^/tbph^Δ23^;repo-GAL4,UAS-GFP/+) and Elav-Repo-Δtb-Gad1 (elav-GAL4,tbph^Δ23^/tbph^Δ23^;repo-GAL4,UAS-GFP/UAS-Gad1). Left graph *n* = 3, right graph *n* = 2. The axis of ordinates showed the ratio between the chosen fragment of the fragmentation spectra of glutamic acid and isotopic labelled glutamic acid. (**b**) Number of peristaltic waves of L3 larvae fed in food containing 50 µM Memantine “D-Drug” or containing the vehicle only “V-Vehicle”, in w^1118^ and ∆tb-23 (tbph^Δ23/Δ23^). *n* = 20. (**c**) Number of peristaltic waves of L3 larvae fed in food containing 5 mM Lithium chloride (LiCl) “D-Drug” or containing the vehicle only “V-Vehicle”, in w^1118^ and ∆tb-23 (tbph^Δ23/Δ23^). *n* = 20. (**d**) Number of peristaltic waves of L3 larvae fed in food containing 50 µM Memantine “D-Drug” or containing the vehicle only “V-Vehicle”, in Elav-Ctrl (UAS-*Dcr-2*/+;elav-GAL4,tbph^Δ23^/UAS-LacZ) and Elav-TBi (UAS-*Dcr-2*/+;elav-GAL4,tbph^Δ23^/+;TBPH-RNAi/+). *n* = 20. (**e**) Confocal images of third instar NMJ terminals in muscle 6/7 second segment stained with anti-HRP (in green) and anti-GluRIIA (in red) in Elav-Ctrl and Elav-TBi with and without the treatment. (**f**) Quantification of GluRIIA intensity. *n* > 200 boutons. ns = not significant *p < 0.05, ***p < 0.001 calculated by one-way ANOVA. Error bars SEM. Scale bar 5 µm.

Alterations in the metabolism of glutamate, the main excitatory neurotransmitter in Drosophila CNS and principal substrate of Gad1, had been previously associated with the neuromuscular defects observed in Gad1 loss-of-function embryos^22^. We therefore investigated whether TBPH minus flies presented altered levels of extracellular glutamate. In these experiments, we quantified the amounts of the neurotransmitter circulating in the hemolymph of mature L3 larvae and found that TBPH mutant flies presented a 1.5-fold increase in the levels of glutamate compared to wild-type controls. Similar results, were obtained upon suppression of Gad1 in Drosophila neurons by RNAi (elav-GAL4/+; GAD-RNAi) (Fig. 4a). To determine whether the excess of glutamate revealed in the extracellular space was causing neurotoxicity in TBPH mutant flies we utilized memantine, a very well characterized glutamate receptor antagonist already used to treat patients with Alzheimer’s disease^28,29^. We found that memantine-fed TBPH minus L3 larvae consistently showed improved movement at the end of the larval period compared to untreated mutants or wild-type controls (Fig. 4b). An identical approach using lithium chloride (LiCl), a less specific antagonist of hyperactivated glutamate receptors, similarly rescued the TBPH minus locomotive problems described in L3 larvae (Fig. 4c). The beneficial effect of these drug treatments became more evident in experiments performed in a TBPH hypomorphic background (Fig. 4d). Indeed, we found that 50μM of memantine administered to flies expressing TBPH-RNAi in neurons (UAS-Dcr-2/+;tbph^∆23^,elav-GAL4/+;TBPH-RNAi/+) was sufficient to allow a complete reconstitution of the GluRIIA fields at the postsynaptic membranes (Fig. 4e,f). This suggested that the excess of glutamate detected in TBPH mutant larvae may have contributed to the neurodegeneration through a persistent activation of their own receptors. Bearing in mind that Gad1 catalyzes the formation of GABA from glutamate, we decided to analyze if defects in the activation of the inhibitory GABAergic pathways contributed to TBPH mutant phenotypes. For these experiments we treated TBPH null flies with different concentrations of GABA^30^ and found that the increase in GABA was unable to modify the reduced locomotive phenotypes of TBPH mutant flies (Supplementary Fig. S5c).

### Gad1 dysregulation is conserved in human cells derived from ALS patients

**Figure 5:**
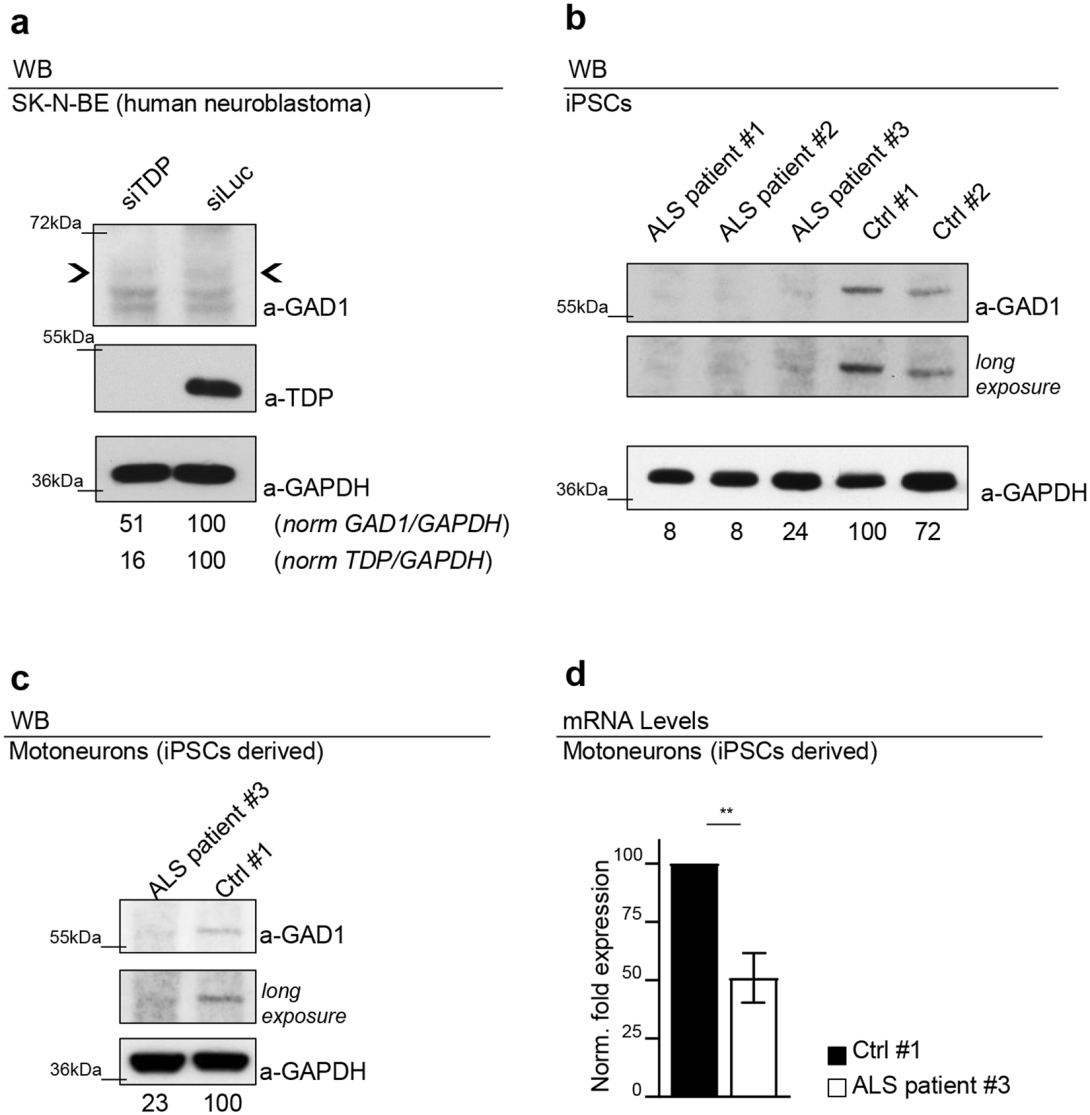
The dysregulation of Gad1 is conserved in human cell lines and iPSCs ALS patients derived. (**a**) Western blot analysis on human neuroblastoma (SK-N-BE) cell line probed for anti-GAD1, anti-GAPDH and anti-TDP in siTDP (TDP silenced) and siLuc (Luciferase ctrl). The same membrane was probe with the three antibodies and the bands of interest were cropped. Quantification of normalized protein amount was reported below each lane, *n* = 3. (**b**) Western blot analysis on human iPSCs probed for anti-GAD1 and anti-GAPDH in three clones derived from three different ALS patients (ALS patient #1 carrying the G287S mutation; ALS patient #2 carrying the G294V mutation; ALS patient #3 carrying the G378S mutation) and in two clones derived from two different healthy subjects (Ctrl #1 Ctrl #2). The same membrane was probe with the two antibodies and the bands of interest were cropped. In the two upper panels were reported two different exposition of anti-GAD1 signal. Quantification of normalized protein amount was reported below each lane. *n* = 3. (**c**) Western blot analysis probed for anti-GAD1 and anti-GAPDH on human differentiated motoneurons derived from iPSCs of an ALS patient (ALS patient #3 carrying the G378S mutation) and a healthy control (Ctrl #1 clone ND41864). The same membrane was probe with the three antibodies and the bands of interest were cropped. In the two upper panels were reported two different exposition of anti-GAD1 signal. Quantification of normalized protein amount was reported below each lane. (**d**) Real time PCR of *Gad1* transcript levels normalized on *Gapdh* (housekeeping) in human differentiated motoneurons derived from iPSCs of an ALS patient (ALS patient #3 carrying the G378S mutation) and a healthy control (Ctrl #1 clone ND41864). *n* = 2, **p < 0.01 calculated by T-test. Error bars SEM.

Interestingly, we found that the suppression of TDP-43 expression in human neuroblastoma SK-N-BE cells induced a similar reduction in the expression levels of GAD67, the Drosophila Gad1 homolog protein, indicating that these modifications were conserved (Fig. 5a and Supplementary Fig. S6b). These results would also suggest that analogous regulatory mechanisms might be affecting ALS patients. To test this hypothesis, we reprogrammed primary fibroblasts from ALS patients containing mutations in TDP-43 (n = 3: patient #1 carrying G287S mutation; patient #2 carrying G294V mutation and patient #3 carrying G378S mutation) to obtain iPSC lines using a protocol based on the transduction of Sendai virus (SeV) vectors containing four reprogramming factors^31^. These vectors are non-integrating and remain in the cytoplasm, allowing the production of iPSCs free of exogenous sequences. iPSC lines reprogrammed from unaffected adult skin fibroblasts (n = 2: control #1 and control #2) were used as controls. Four weeks after reprogramming, colonies with pluripotent morphology were selected for further expansion and analysis. We found that in iPS cells derived from ALS patients, the levels of GAD67 protein resulted clearly downregulated or even absent when compared with the two controls (Fig. 5b and Supplementary Fig. S6c). In addition, ALS iPSCs (from patient #3) and iPSCs from control (#1) were differentiated into spinal motor neurons (MNs) using a multistep protocol already described for human iPSCs^32^. We performed western blot analysis to evaluate the expression of GAD67 in ALS MNs compared to control and we confirmed a clear downregulation of GAD67 protein also in MNs (Fig. 5c and Supplementary Fig. S6d). Remarkably, quantitative PCR analysis revealed that the levels of GAD67 mRNA were also reduced in MNs (Fig. 5d) strongly supporting a preserved role for this enzyme in the neurodegenerative process behind ALS.

## Discussion

Alterations in motoneuronal excitability derived from defects in the activity of strategic enzymes required for the synthesis and metabolism of the neurotransmitters have been previously associated with the pathogenesis of ALS^18,33,34^. However, the interpretation of the results reported has always proved difficult due to the lack of experimental evidence connecting the molecular modifications observed with the physiopathogenesis of the disease. To fill this gap, we utilized the Drosophila system in which we have previously described that the suppression of the TDP-43 homolog protein closely reproduces the molecular characteristics observed in ALS^14^. Subsequently, our analysis showed that Gad1, the enzyme catalyzing the decarboxylation of glutamate to GABA, appeared downregulated in TBPH minus fly heads compared to wild-type controls and demonstrated that the genetic rescue of Gad1 deficiencies in TBPH-null presynaptic neurons or glia was sufficient to recover flies motility, extracellular glutamate levels and the postsynaptic distribution of the glutamate receptors at the neuromuscular junctions. Moreover, we found that defects in Gad1 levels associated with pathological modifications in TDP-43 were conserved in human cell lines and present in iPSC-derived motoneurons from ALS patients, linking these events with the potential mechanisms of the disease.

### Defects in Gad1 activity due to TBPH loss induced glutamate excitotoxicity in flies

Although it is commonly accepted that excitotoxicity plays an important role in the pathogenesis of ALS, it was not clear so far whether brains affected by TDP-43 dysfunctions present alterations in the metabolism of glutamate or if these alterations are neurotoxic. In this respect, we found that null alleles of TBPH show increased levels of circulating glutamate in the hemolymph. Perhaps more interestingly, these results were confirmed by the suppression of Gad1 activity exclusively in neurons by expression of a specific RNAi against the enzyme. Moreover, we found that TBPH minus flies showed an increased sensitivity to the neurotransmitter (Supplementary Material, Fig. S5b)^30^, confirming the incidence of a higher glutamate signaling in these flies. In agreement with this hypothesis, we reported that memantine, a specific glutamate receptor antagonist, significantly improved the locomotive ability of TBPH-null larvae and stimulated the postsynaptic recovery of the glutamate receptors in the NMJs of TBPH-minus hypomorphic alleles (Fig. 4b,e,f). These results indicate that TBPH function is necessary to prevent glutamatergic excitotoxicity by maintaining normal levels of Gad1 activity. In agreement with this interpretation, previous experiments performed in Drosophila embryos had shown that Gad1 activity affects the postsynaptic organization of glutamate receptors at neuromuscular junctions through the regulation of the neurotransmitter release^22^.

Considering that Gad1 activity is also required to synthesize GABA, we analyzed whether defects in this neurotransmitter played any role in the neurological phenotypes caused by the absence of TBPH. We found that the administration of GABA (as described in Drosophila models of Fragile X syndrome^30^) was ineffective to reverse the locomotive alterations described in TBPH minus alleles (Supplementary Fig. S5c). Alike, the administration of the GABA_B_ antagonist CGP55845^30^ was unable to modify the TBPH minus phenotypes (Supplementary Fig. S5d). Although, these experiments did not allow to exclude the eventual role of the GABA signaling in TBPH minus defects, our results support the notion that the excessive production of glutamate was behind these neurological alterations.

### How does TBPH regulate Gad1 levels?

Despite, the strong genetic and biochemical correlations defined in this study, we did not detect direct physical interactions between TBPH and the messenger of Gad1 by co-immunoprecipitation^17,21^, suggesting that these regulatory mechanisms may involve some other intermediate molecules. Concerning this issue, TBPH regulates many different targets and is involved in numerous aspects of RNA metabolism through specific RNA-binding proteins some of which may be directly influencing the stability or expression levels of Gad1. Indeed, we observed that Gad1 mRNA levels were affected by the absence of TBPH indicating that transcription or splicing defects may have a role in the downregulation of this enzyme^35–37^. Alternatively, alterations in synaptic transmission, cytoplasmic transport or autophagy induced by the absence of TBPH could affect the vesicular localization of Gad1, its intracellular localization or its activity^38–40^. Defects in TBPH could also downregulate Gad1 through the activation of stress responsive pathways that mediate the enzymatic cleavage and degradation of this protein when cells go to apoptosis^40^. Additional experiments would be required to address these issues.

### The association between Gad1 levels and TDP-43 dysfunction is conserved

Defects in the human Gad1 homolog proteins GAD67 and GAD65 were found in patients suffering from different neurodegenerative diseases including Parkinson, Huntington and Alzheimer’s^41–43^ or cognitive disorders like idiopathic autisms or schizophrenia^44–46^, suggesting that a tight regulation of this enzyme is critical to preserve normal brain functioning. In the case of ALS, different lines of evidence suggest that glutamate-induced cytotoxicity might play an important role in the development of the disease^33^. Interestingly, we found that the reduction of TDP-43 expression in human neuroblastoma cells induced similar modifications in the protein levels of the human homolog protein GAD67. More strikingly, we found identical modifications in iPSC-derived motoneurons obtained from patients carrying TDP-43 mutations and suffering from the disease, indicating that these regulatory mechanisms are conserved. Analogously, our results imply that similar defects in the regulation of the neurotransmitter could disturb the synaptic organization of motoneuron terminals in affected patients and suggest that the pharmacological modulation of the glutamate receptors or an excessive glutamate signaling might be beneficial to limit the neurodegenerative process in ALS.

## Material and Methods

### Fly strains

The genotype of the flies used in this work are indicated below:

w^1118^ - w;tbph^Δ23^/CyO^GFP^ - w;tbph^Δ142^/CyO^GFP^ - w;elav-GAL4/CyO^GFP^ - w;D42-GAL4 - repo-GAL4/TM3,Sb - w;;tubulin-GAL4/TM2 - w;tubulin-GAL80^TS^ - UAS-Dcr-2 - gliotactin-GAL4/CyO^GFP^ (gifted by Dr. Vanessa Auld) - w;UAS-mCD8::GFP - w;UAS-TBPH - w;Mi[MIC]Gad1^MI09277^/TM3,Sb,Ser - UAS-Gad1/TM3,Sb (gifted by Dr. Andreas Prokop) - UAS-GAD-RNAi/TM3Sb (#28079, Bloomington) - UAS-TBPH-RNAi/TM6b (#ID38377, VDRC) - UAS-LacZ/CyO.

### Larval movement and Climbing assay

The protocol previously described in^14^ has ben used.

### Drug treatment

Memantine 4.6 mM, Lithium chloride 1 M, GABA 8 M and CGP55845 100 mM were prepared and then were diluted during the fly food preparation in accordance to the final concentration required. Control groups have been fed in a standard food containing vehicle only. Parental flies have been maintained 24 hours in the tubes to allow the embryo laying. Embryos were grown to obtain third instar larvae used for the further analysis.

### Hemolymph extraction and sample preparation

Hemolymph was pooled from 15–18 third instar larvae to obtain a final volume of 2 µl. The two extremities of larvae were pinned, then transferred in a 0.5 ml tube with a 3 mm cut on the bottom and inserted in a 1.5 ml tube. Tubes were centrifuged at top speed for 20 seconds and equal volumes of the hemolymph drop in the bottom of 1.5 ml tube were immediately collected in a new tube and stored at −80 °C until analysis.

4 μl of the hemolymph and standard samples, were diluted with 200 μl of internal standard solution (10µM L-glutamic acid-2,3,3,4,4-d_5_, Merck KGaA, Germany) in 60% acetonitrile - 40% water with 10 mM NH_4_OAc. After centrifugation, 5 μl of this solution were injected into the LC–MS/MS.

### Gradient elution profile and instruments

Mobile phase “A” and “B” consisted of 0.1% formic acid in water (LC–MS grade) and 0.1% formic acid in acetonitrile (LC–MS grade), respectively. The gradient elution profile was chosen as follows: 0 min: 10% A, 3 min: 10% A, 13.00 min: 100% A, 20 min: 100% A, 21 min: 10% A, 31 min: 10% A. The HPLC system consisted of a Gilson 234 autosampler (Gilson Inc., Middleton, WI), Gilson 306 Binary Pump, and a Thermostatted Column Compartment (Agilent Technologies, Morges, Switzerland). Chromatographic retention was obtained using a hydrophilic interaction liquid chromatography (HILIC) column (ZIC®-HILIC, 150 × 2.1 mm i.d., 3.5μm, Merck KGaA, Germany) at 30 °C run at 250ul/min. Eluates were detected using an amaZonSL ion trap mass spectrometer (Bruker Daltonik GmbH, Germany) in the positive electrospray ionisation (ESI) mode. The ion spray voltage was set at 4500 V and the source temperature at 200 °C. Data were acquired monitoring the m/z 148.1/84.1 transition (L-Glu) and the 153.1/89.1 transition (d5-L-Glu internal standard) and analyzed using the software Compass Data Analysis 4.2 (Bruker).

### Immunohistochemistry

The neuromuscular junctions of third instar larvae were dissected as described in^17^. Dilutions of antibodies used are reported here: anti-HRP (Jackson 1:150), anti-GFP (Life Technologies 1:200), anti-GluRIIA 8B4D2c (DSHB 1:15), anti-Dlg 4F3c (DSHB 1:250), anti-Syx 8C3s (DSHB 1:15), anti-Bruchpilot (DSHB 1:50), anti-HRP-Cy3 (Jackson 1:150), Alexa-Fluor® 488 (mouse or rabbit 1:500) and Alexa- Fluor® 555 (mouse and rabbit 1:500).

### Acquisition and quantification of confocal images

In each experiment, the genotypes of interest were processed simultaneously and the images were acquired using the same settings. Images of muscle 6 and 7 on second abdominal segment were acquired using the LSM Zeiss Software on a Zeiss 510Meta confocal microscope (63 × oil lens) and then analyzed using ImageJ (Wayne Rasband, NIH). For the quantification of pre and postsynaptic markers, samples were double labelled with anti-HRP and the marker of interest: the ratio between the mean intensity of the marker and the HRP was calculated for each bouton of the terminal. The quantification of glial area was calculated analyzing the ratio between the area occupied by glial tissue versus the area of the presynaptic terminal^17,18^.

### Bi-dimensional protein separations (IEF/SDS-PAGE) and mass-spectrometry analysis

Three independent fly head protein extractions were performed, and protein concentrations were evaluated by Bradford. Protein extractions were TCA precipitated, solubilized in isoelectrofocusing (IEF) rehydration solution containing 0.6% IPG buffer pH 3-11 NL (GE Healthcare Bio-sciences AB) and separated according to their isoelectric point on nonlinear immobilized pH-gradient strips (pH3-11 NL) with a length of 13 cm. The isoelectrofocusing program was: 50 V for 4 hours, 500 V for 1 hour, 1,000 V for 2 hours, and 8,000 V up to 48,000 V × h. Focused strips were equilibrated with 2.5 ml of the following solutions: 6 M urea, 30% glycerol, 50 mM Tris–HCl (pH 8.8), 2% SDS, 25 mg DTT and bromophenol blue; then 6 M urea, 30% glycerol, 50 mM Tris–HCl (pH 8.8), 2% SDS, 62.5 mg iodoacetamide and bromophenol blue. The strips were subjected to electrophoretic separation on 14% SDS-polyacrilamide gels (18 × 20 × 0.15 cm) run at 12 mA O.N. The gels were then fixed and stained (0.12% Colloidal G-250 Coomassie Blue). Spot intensity acquisitions were performed at 300 dpi and in conditions of linear response in an Epson Expression 1680 Pro scanner. Values of pI were calculated according to the pH curve of the pH gradient strips provided by the supplier (GE Life Sciences), while protein molecular masses were calculated from the semi-logarithmic curves of log_10_ molecular mass versus migration distance. The images acquired for each of the gels (triplicates) were analyzed with the Redfin 3 program (Ludesi AB, Lund, Sweden) which includes all-to-all spot matching and normalization according to the total protein content, taken as the sum of the intensities of all the spots contained in each gel. Proteins were considered to be differentially expressed when their mean spot intensity differed by >1.5-fold between groups, with *p* < 0.05. Relevant spots were excised and sent to the proteome and mass spectrometric service Proteome Factory AG (Proteome Factory AG, Germany) for nanoLC-ESI-MS/MS analysis.

### Western blot on Drosophila brains, transfected neuroblastoma SK-N-BE, and iPSC lines

Drosophila larval brains were homogenized in lysis buffer (10 mM Tris, 150 mM NaCl, 5 mM EDTA, 5 mM EGTA, 10% Glycerol, 50 mM NaF, 5 mM DTT, 4 M Urea, pH 7.4, plus Protease Inhibitors (Roche #11836170001)).

SK-N-BE and iPS cell lines, were resuspended in iced PBS plus Protease Inhibitors and subjected to sonication (Biorupture, Diagenode).

Lysates were quantified (#Q33211, Invitrogen), separated on SDS-PAGE and transferred to nitrocellulose membrane (#NBA083C Whatman Protran). Blots were blocked with 5% non-fat dry milk or 5% BSA, if required by antibody supplier, in TBS-T (Tris-buffered saline, 0.1% Tween 20).

The panel of primary antibodies used includes: anti-Flag M5 (F4042 Sigma 1:10,000), GAD1 (#5305 Cell Signaling 1:2,000), anti tubulin DM1A (#CP06 Calbiochem 1:5,000) and the anti GAPDH (#sc-47724 Santa Cruz 1:2,000). Secondary antibody incubation was performed with anti-mouse HRP conjugated or anti-rabbit HRP conjugated (#32430; #32460 Pierce 1:1000). SuperSignal West Femto Maximum Sensitivity Substrate Kit (#PR34095 Pierce) was used for detection and protein quantification was performed using ImageJ.

### RT-PCR

Third instar larval brains and motoneurons were mechanically squeezed to proceed with RNA extraction, using RNeasy®Microarray MiniKit (50) (#73304 Qiagen) and the QIAshredder^TM^ kit(50) (#79654 Qiagen). The RNA was than treated with DNase (#M6101 Promega) and oligo-dT retro-transcripted with SuperScript® III First-Strand Synthesis (#18080051 ThermoFisher). Gene specific primers were designed for amplification:

Gad1: 5′*GAATTCAGAGCATAGAAGCCACCG3*′ *and* 5′*CAACTGGCTCTTCATCTTCTCCTG3*′

Rpl11 5′*CCATCGGTATCTATGGTCTGGA3*′ *and* 5′*CATCGTATTTCTGCTGGAACCA3*′

Gapdh: *5*′*CTGGGCTACACTGAGCACC3*′ *and 5*′*AAGTGGTCGTTGAGGGCAATG3*′

Gad67: *5*′*CCTCAACTATGTCCGCAAGAC3*′ *and 5*′*TGTGCGAACCCCATACTTCAA3*′

The quantification was calculated according the ΔΔC_T_ equation and then normalized on control genotype.

### Cell culture and RNA interference

SK-N-BE neuroblastoma cell line was cultured in standard conditions in DMEM-Glutamax (#31966-021, Thermo Fisher Scientific) supplemented 10% fetal bovine serum and 1 × antibiotic-antimycotic solution (#A5955; Sigma). RNA interference of TDP-43 was achieved using HiPerfect Transfection Reagent (#301705, Qiagen) and siRNA specific for human TDP43 (5′-gcaaagccaagaugagccu-3′); as control siRNA for Luciferase was used (5′-uaaggcuaugaagagauac-3′; Sigma). Immediately before transfection 2–4 × 10^5^ cells were seeded in 6-well plates in 1.4 ml of medium containing 10% fetal serum. A volume of 3 µl of each siRNA (40 µM solution in water), was added to 91 µl of Opti-MEM I reduced serum medium (#51985-026, Thermo Fisher Scientific), incubated 5 minutes at room temperature and subsequently 6 µl of HiPerfect Transfection Reagent were added. The silencing procedure was performed again after 24 and 48 hours.

### Human iPSC Culture and MN differentiation

The studies involving human samples were conducted in compliance with the Code of Ethics of the World Medical Association (Declaration of Helsinki) and with national legislation and institutional guidelines. Fibroblasts were generated from dermal biopsies (Eurobiobank) following informed consent (ethical committee approved at the IRCCS Foundation Ca’ Granda Ospedale Maggiore Policlinico). Fibroblasts from dermal biopsies of ALS patients (n = 3: patient #1 carrying G287S mutation; patient #2 carrying G294V mutation and patient #3 carrying G378S mutation) and control subjects (n = 2: control #1 and control #2) were reprogrammed into iPSCs using CytoTune®-iPS 2.0 Sendai Reprogramming Kit (Life Technologies)^31^, containing Sendai virus (SeV) vectors in which four reprogramming factors (OCT4, SOX2, c-Myc, and KLF4) were cloned. iPSC colonies with embryonic stem cell (ESC)-like morphology were cultured and expanded on Matrigel-coated dishes (BD Biosciences) in Essential E8 media (Life Technologies). All cell cultures were maintained at 37 °C, 5% CO_2_.

iPSCs were differentiated into MNs using a multistep protocol as described^32^.

### Statistical analysis

The statistical analysis was performed with Prism (GraphPad, USA) version 7.0.

### Data Availability

The datasets generated during and/or analysed during the current study are available from the corresponding author on reasonable request.

## Electronic supplementary material

Supplementary Material
