# Supplementary material for: Downregulation of glutamic acid decarboxylase in Drosophila TDP-43-null brains provokes paralysis by affecting the organization of the neuromuscular synapses
Source: Sci Rep. 2018 Jan 29;8:1809. doi: 10.1038/s41598-018-19802-3 (PMC5789004; doi:10.1038/s41598-018-19802-3)
Supplement: Supplementary file 1 — Supplementary Material [file 41598_2018_19802_MOESM1_ESM.pdf]

SUPPLEMENTARY MATERIAL

Downregulation of glutamic acid decarboxylase in Drosophila TDP-43-null brains provokes paralysis by affecting the organization of the neuromuscular synapses

Giulia Romano[1], Nikola Holodkov[1], Raffaella Klima[1], Federica Grilli[1], Corrado Guarnaccia[1], Monica Nizzardo[2], Federica Rizzo[2], Rodolfo Garcia[1] and Fabian Feiguin[1*]

[1] International Centre for Genetic Engineering and Biotechnology, Padriciano 99, 34149 Trieste, Italy.

[2] Dino Ferrari Centre, University of Milan, Department of Pathophysiology and Transplantation (DePT), Neuroscience Section, IRCCS Foundation Ca' Granda Ospedale Maggiore Policlinico, Via Francesco Sforza 35, 20122, Milan, Italy.

[*] Corresponding author

E-mail: fabian.feiguin@icgeb.org
Phone:+39-040-3757201
Fax: +39-040-226555

Supplementary figure 1

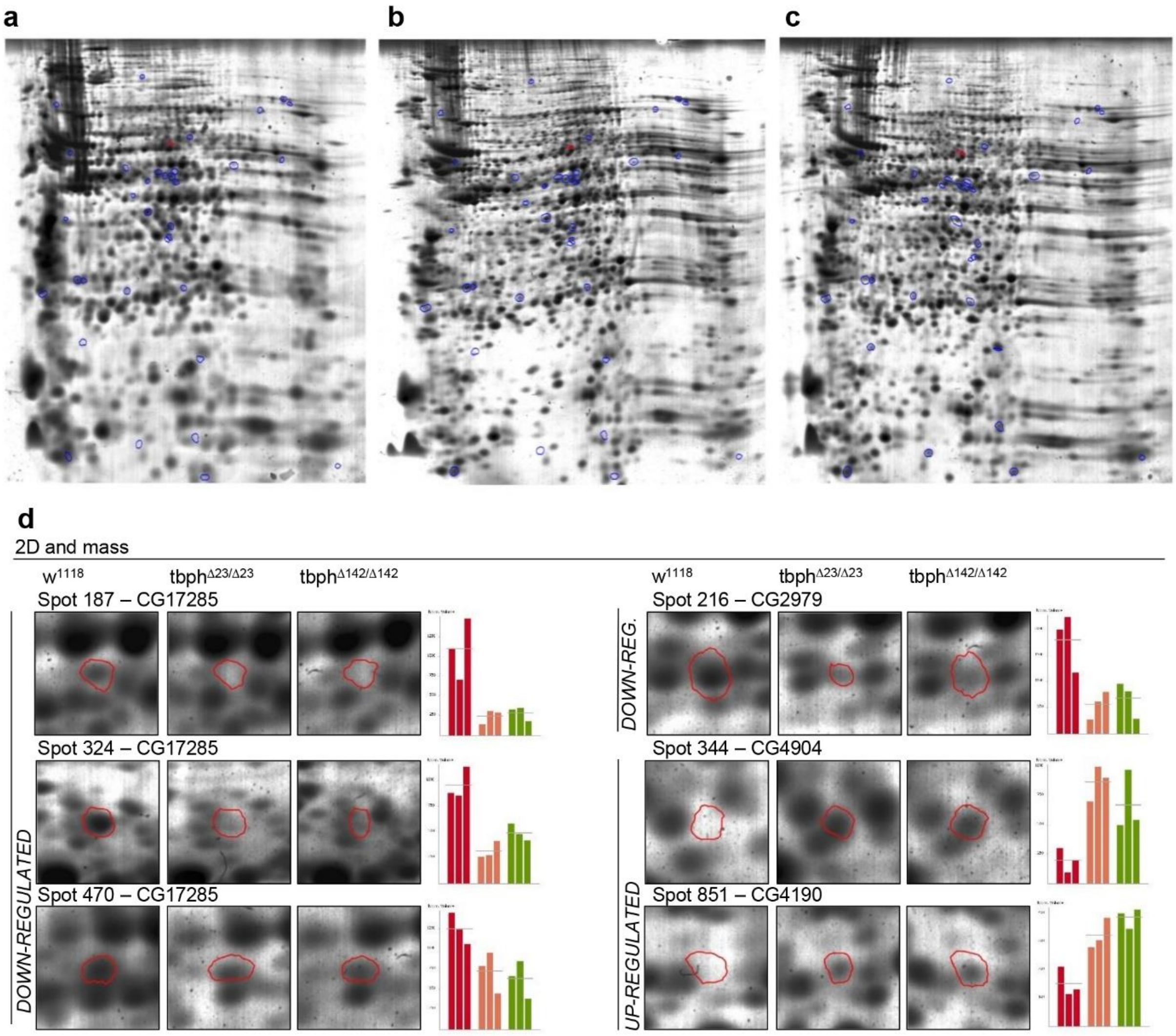

Supplementary figure 2

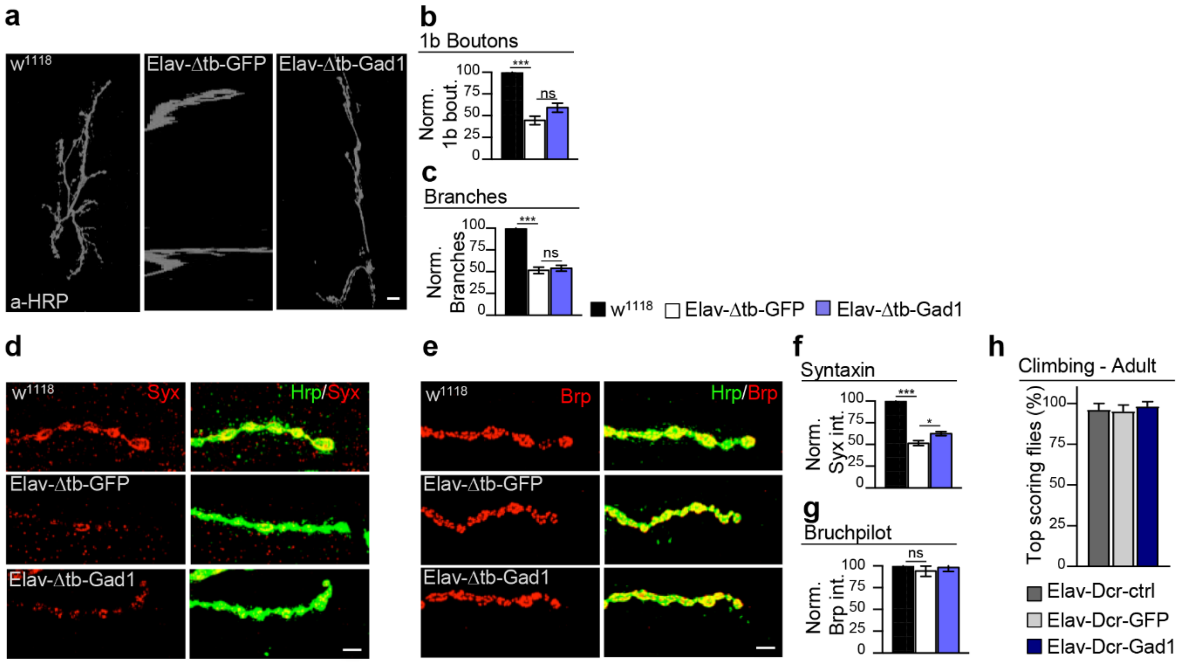

Supplementary figure 3

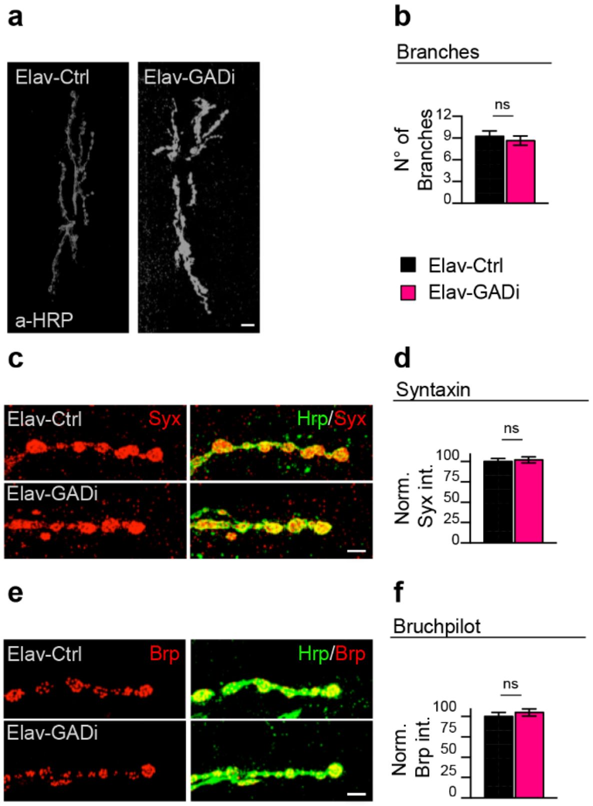

Supplementary figure 4

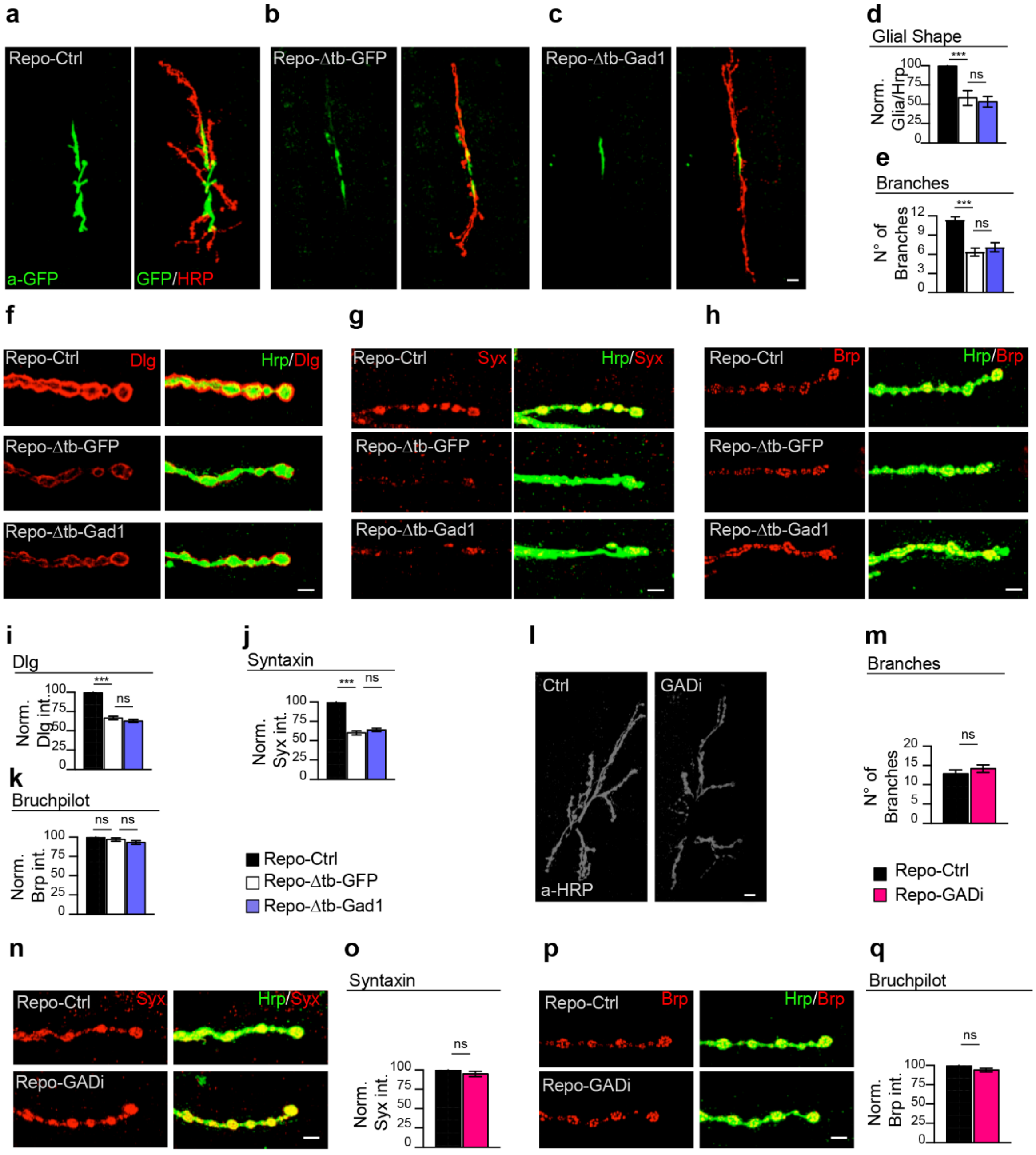

Supplementary figure 5

a

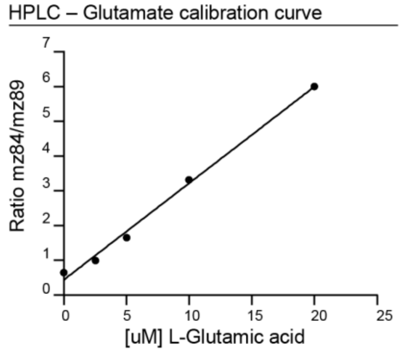

b

Eclosion rate □ food+vehicle ■ food+Glutamate 80mM

	Eclosed	Trapped	Dead
tbph$^{\Delta23}$/CyO^{GFP}	91%	3%	6%
tbph$^{\Delta23}$/CyO^{GFP}	89%	2%	9%
tbph$^{\Delta23/\Delta23}$	47%	34%	19%
tbph$^{\Delta23/\Delta23}$	27%	40%	33%

c **d** **e**

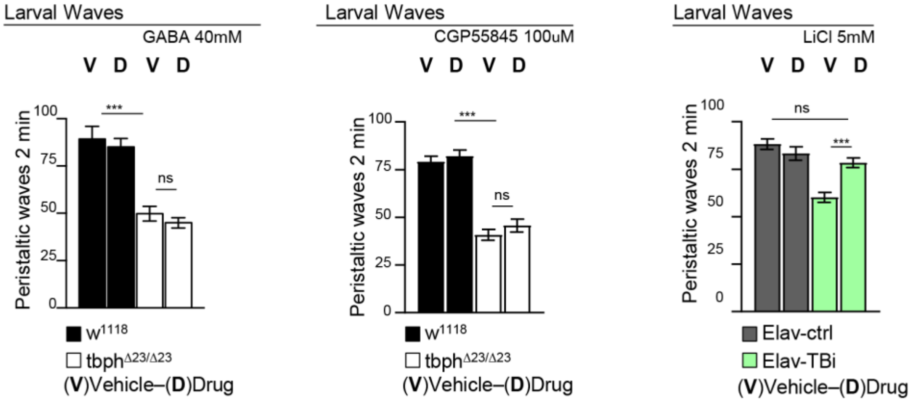

Supplementary figure 6

a

Complete membrane Fig. 1b

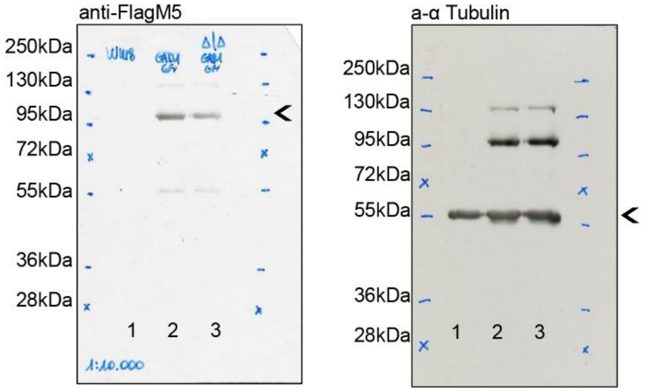

c

Complete membrane Fig. 5b

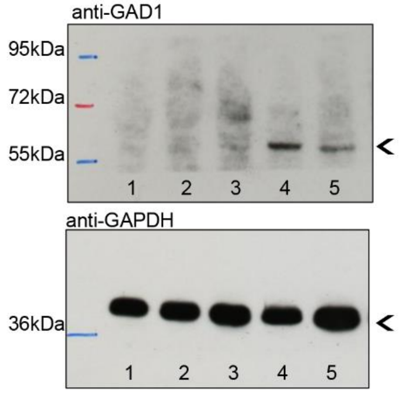

b

Complete membrane Fig. 5a

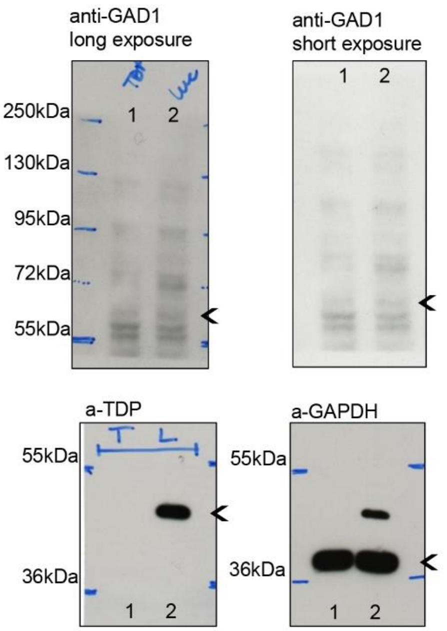

d

Complete membrane Fig. 5c

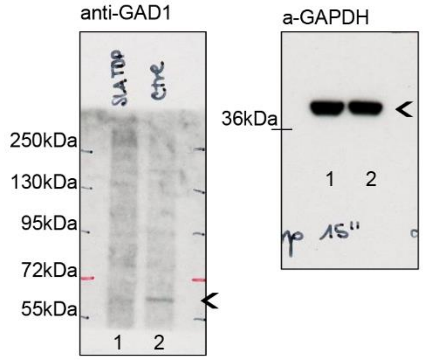

Supplementary Figure Legends

Supplementary figure 1. 2D gels and spots modified.

(**a-c**) 2D gels showed the 35 modified spots (blue circle), in the red circle there is Spot 701 corresponding to Gad1 respectively of w^{1118} (a), $tbph^{\Delta23/\Delta23}$ (b) and $tbph^{\Delta142/\Delta142}$ (c). *n*=3 (**d**) Sections of the gels containing the 5 down-regulated and the 2 up-regulated spots in both TBPH mutants ($tbph^{\Delta23}$ and $tbph^{\Delta142}$) compare to w^{1118}. Bar graph on the right show the normalized intensities corresponding to each spot. *n*=3.

Supplementary figure 2. The recovery of Gad1 activity does not promote presynaptic growth.

(**a**) Confocal images of third instar NMJ terminals in muscle 6/7 second segment stained with anti-HRP in w^{1118}, Elav-Δtb-GFP (elav-GAL4,$tbph^{\Delta23}$/$tbph^{\Delta23}$;UAS-GFP/+) and Elav-Δtb-Gad1 (elav-GAL4,$tbph^{\Delta23}$/$tbph^{\Delta23}$;UAS-Gad1/+). (**b-c**) Quantification of 1b boutons and quantification of synaptic branches present on muscle 6/7, second segment. *n*=15 larvae. (**d**) Confocal images of third instar NMJ terminals in muscle 6/7 second segment stained with anti-HRP (in green) and anti-Syntaxin (in red) in w^{1118}, Elav-Δtb-GFP and Elav-Δtb-Gad1. (**e**) Confocal images of third instar NMJ terminals in muscle 6/7 second segment stained with anti-HRP (in green) and anti-Bruchpilot (in red) in w^{1118}, Elav-Δtb-GFP and Elav-Δtb-Gad1. (**f-g**) Quantification of Syntaxin intensity and Bruchpilot intensity. *n*>200 boutons. (**h**) Climbing assay on adult flies at day 4 of Elav-Dcr-ctrl (elav-GAL4,$tbph^{\Delta23}$/+;UAS-*Dcr-2*/+), Elav-Dcr-GFP (elav-GAL4,$tbph^{\Delta23}$/UAS-GFP;UAS-*Dcr-2*/+) and Elav-Dcr-Gad1 (elav-GAL4,$tbph^{\Delta23}$/+;UAS-*Dcr-2*/UAS-Gad1). *n*=100. ns=not significant *p<0.05, **p<0.01, ***p<0.001 calculated by one-way ANOVA. Error bars SEM. Scale bar 10µm (a) and 5µm (d and e).

Supplementary figure 3. The suppression of Gad1 activity in neurons does not affect presynaptic organization.

(**a**) Confocal images of third instar NMJ terminals in muscle 6/7 second segment (AII) stained with anti-HRP in Elav-Ctrl (UAS-LacZ/elav-GAL4) and Elav-GADi (elav-GAL4/+;UAS-GAD-RNAi/+). (**b**) Quantification of synaptic branches. *n*=15 larvae. (**c**) Confocal images of third instar NMJ terminals in muscle 6/7 second segment stained with anti-HRP (in green) and anti-Syntaxin (in red) in Elav-Ctrl and Elav-GADi. (**d**) Quantification of Syntaxin intensity in Elav-Ctrl and Elav-GADi. *n*=200 boutons. (**e**) Confocal images of third instar NMJ terminals in muscle 6/7 second segment stained with anti-HRP (in green) and anti-Bruchpilot (in red) in Elav-Ctrl and Elav-GADi. (**f**) Quantification of Bruchpilot intensity in Elav-Ctrl and Elav-GADi. *n*=200 boutons. ns=not significant, calculated by T-test. Error bars SEM. Scale bar 10µm in (a) and 5µm in (c and e).

Supplementary figure 4. The glial activity of Gad1 does not promote axonal wrapping.

(**a-c**) Confocal images of third instar NMJs expressing mCD8-GFP in glial cells using repo-GAL4 double labeled with anti HRP (in red) and anti-GFP (in green) in (a) Repo-Ctrl ($tbph^{\Delta23}$/+;repo-GFP,UAS-GFP/+), (b) Repo-Δtb-GFP ($tbph^{\Delta23}$/$tbph^{\Delta23}$;repo-GFP,UAS-GFP/+) and (c) Repo-Δtb-Gad1 ($tbph^{\Delta23}$/$tbph^{\Delta23}$;repo-GFP,UAS-GFP/UAS-Gad1). (**d**) Quantification of glial area. *n*=15 larvae. (**e**) Quantification of synaptic branches present on muscle 6/7, second segment. *n*>15 larvae. (**f**) Confocal images of third instar NMJ terminals in muscle 6/7 second segment stained with anti-HRP (in green) and anti-Dlg (in red) in Repo-Ctrl, Repo-Δtb-GFP and Repo-Δtb-Gad1. (**g**) Confocal images of third instar NMJ terminals in muscle 6/7 second segment stained with anti-HRP (in green) and anti-Syntaxin (in red) in Repo-Ctrl, Repo-Δtb-GFP and Repo-Δtb-Gad1. (**h**) Confocal images of third instar NMJ terminals in muscle 6/7 second segment stained with anti-HRP (in green) and anti-Bruchpilot (in red) in Repo-Ctrl, Repo-Δtb-GFP and Repo-Δtb-Gad1. (**i-k**) Quantification of Dlg intensity, Syntaxin intensity and Bruchpilot intensity. *n*>200 boutons. (**l**) Confocal images of third instar NMJ terminals in muscle 6/7 second segment stained with anti-HRP in Repo-Ctrl (UAS-*Dcr-2*/+;UAS-LacZ/+;repo-GAL4/+) and Repo-GADi (UAS-*Dcr-2*/+;+/+;repo-GAL4/GAD-RNAi). (**m**) Quantification of synaptic branches present on muscle 6/7, second segment. *n*=15 larvae. (**n**) Confocal images of third instar NMJ terminals in muscle 6/7 second segment stained with anti-HRP (in green) and anti-Syntaxin (in red) in Repo-Ctrl and Repo-GADi. (**o**) Quantification of Syntaxin intensity. *n*>200 boutons. (**p**) Confocal images of third instar NMJ terminals in muscle 6/7 second segment stained with anti-HRP (in green) and anti-Bruchpilot (in red) in Repo-Ctrl and Repo-GADi. **(q)** Quantification of Bruchpilot intensity. *n*>200 boutons. ns=not significant, ***p<0.001, calculated by one-way ANOVA in (d,e,i,j and k) and T-test in (m,o and q). Error bars SEM. Scale bar 10µm in (a-c and l) and 5µm in (f,g,h,n and p).

Supplementary figure 5. High glutamate and not GABA affect locomotive behaviors in TBPH-null flies.

(**a**) Calibration curve by plotting peak area ratio (L-Glu/d_5 Glu; mz84/mz89) as a function of concentration for a series of known concentrations of L-Glutamic acid. Linear regression analysis, R square=0.9947, y=0.2778x+0.4406. (**b**) Eclosion analysis of larvae fed in food containing 80mM Glutamate versus flies fed in normal food (vehicle) in $tbph^{\Delta23}$/CyO and $tbph^{\Delta23/-}$. The number of the eclosed flies, trapped in the cage and dead pupae were counted for each genotype and percentage reported in the graph. *n*=100. (**c**) Number of peristaltic waves of L3 larvae fed in food

containing 40mM GABA "D-Drug" or containing the vehicle only "V-Vehicle", in w^{1118} and $tbph^{\Delta23/\Delta23}$. *n*=20. (**d**) Number of peristaltic waves of L3 larvae fed in food containing 100uM CGP55845 "D-Drug" or containing the vehicle only "V-Vehicle", in w^{1118} and $tbph^{\Delta23/\Delta23}$. *n*=15. (**e**) Number of peristaltic waves of L3 larvae fed in food containing 5mM Lithium chloride "D-Drug" or containing the vehicle only "V-Vehicle", in Elav-Ctrl (UAS-*Dcr-2*/+;elav-GAL4,$tbph^{\Delta23}$/UAS-LacZ) and Elav-TBi (UAS-*Dcr-2*/+;elav-GAL4,$tbph^{\Delta23}$/+;TBPH-RNAi/+). *n*=20. ns=not significant, ***p<0.001, calculated by one-way ANOVA. Error bars SEM.

Supplementary figure 6. Full length protein gels.

(**a**) Full length membrane of Fig. 1c. Western blot analysis of third instar larval brains probed with the anti-FlagM5 on the left panel and on the right panel with the anti- alpha-tubulin antibodies. Genotypes lane 1= w^{1118}; lane 2= MiMIC tagged endogenous Gad1 in wild-type and TBPH-null backgrounds (w;Mi{MIC}$Gad1^{MI09277}$/+); lane 3= $Gad1^{MiMIC}$- Δtb ($tbph^{\Delta23}$/$tbph^{\Delta23}$;Mi{MIC}$Gad1^{MI09277}$/+). (**b**) Full length membrane of Fig. 5a. Western blot analysis on human neuroblastoma (SK-N-BE) cell line probed probed with anti-GAD1 (right and left upper panel), anti-GAPDH (right lower panel) and anti-TDP (left lower panel) in lane 1=siTDP (TDP silenced) and lane 2= siLuc (Luciferase ctrl). (**c**) Full length membrane of Fig. 5b. Western blot analysis on human iPSCs probed for anti-GAD1 and anti-GAPDH in three clones derived from three different ALS patients (lane 1= ALS patient #1 carrying the G287S mutation; lane 2= ALS patient #2 carrying the G294V mutation; lane 3= ALS patient #3 carrying the G378S mutation) and in two clones derived from two different healthy subjects (lane 4= Ctrl #1 and lane 5= Ctrl #2). (**d**) Full length membrane of Fig. 5c. Western blot analysis probed for anti-GAD1 and anti-GAPDH on human differentiated motoneurons derived from iPSCs of an ALS patient (lane 1= ALS patient #3 carrying the G378S mutation) and a healthy control (lane 2= Ctrl #1 clone ND41864).

Supplementary Material and Methods

Fly maintenance

The fly strains were maintained at 25°C on a 12:12h light:dark cycle, at constant 60% humidity on a standard cornmeal medium (agar 6.25g/l, yeast 62.5g/l, sugar 41.6g/l, corn flour 29g/l, 4.1ml propionic acid).

Eclosion analysis

Embryos from tbph$^{\Delta23}$/CyO^{GFP} were collected for 2 hours in tubes containing food+vehicle or food+80mM Glutamate at 25°C. Homo and heterozygous larvae were separated at the final L3 stage and placed (at same population concertation) in fresh tubes to evaluate the rate of the eclosed, trapped and dead flies.

MNs differentiation from iPSCs

iPSC colonies were detached and put in suspension to form Embryo Bodies (EBs) in E8 medium for seven days. For the first three days, cells were kept in suspension in E8 medium, supplemented with Y27632 (Ascent Scientific 10µM), bFGF (Life Technologies 20ng/mL), SB435142 (Sigma 10µM) and LDN193189 (Stemgent 0.2µM). At day 3, EBs were switched to neural induction medium (DMEM/F:12 with L-glutamine, NEAA, penicillin/streptomycin, heparin 2µg/ml), N2 supplement (Life technologies). At day 5, retinoic acid (Sigma 1µM), ascorbic acid (Sigma 0.4 µg/ml), and BDNF (R&D 10ng/mL) were added. At day 7, hedgehog signaling was started adding Sonic Hedgehog (Sigma 200ng/ml). At day 17, basal medium was changed to neural differentiation medium (Neurobasal with L-glutamine, MEM, N2 supplement), containing previous factors and with the addition of 10ng/mL each of IGF-1, GDNF, and CNTF (R&D), plus B27 (Life technologies). At day 20, EBs were dissociated with 0.05% trypsin (Sigma), and plated onto poly-lysine/laminin-coated dishes (BD Biosciences). Plated neurons were cultured in the same medium with the addition of BME (Sigma 25µM), and glutamic acid (Sigma 25µM), changing the medium two times a week. At day 40, the cells were harvested for western blot analysis.
